# Advances in Functional Polymers and Nanocomposites (Second Edition)

**DOI:** 10.3390/ma19051030

**Published:** 2026-03-08

**Authors:** Barbara Gawdzik, Przemysław Pączkowski

**Affiliations:** Department of Polymer Chemistry, Institute of Chemical Sciences, Faculty of Chemistry, Maria Curie-Skłodowska University, Gliniana 33, 20-614 Lublin, Poland; przemyslaw.paczkowski@umcs.pl

Conventional polymers are materials used in everyday life in the form of plastics, polymer composites, synthetic fibers, paints and coatings, etc. However, with the development of modern society, certain areas require special properties that conventional polymers do not possess. However, developments in the field of polymer materials are proceeding at an extremely rapid pace, and innovative solutions are enabling the creation of new polymers with specific functions.

Functional polymers are polymeric materials with one or more specific chemical groups in their structure.

Polymers can be endowed with functions through special preparation methods, by incorporating functional groups into their structures through chemical modifications or suitable fillers. These functions are either inherent to the materials or can be activated by external stimuli. Polymers prepared in this way exhibit interesting physical, biological, pharmacological, and other properties [1]. Functional polymers find applications in numerous fields, such as drug delivery, tissue engineering, medical devices, food packaging, sensors, optoelectronics, organic light-emitting diodes, batteries, and wastewater treatment [2,3,4,5,6,7,8,9,10,11,12,13,14].

It is important to remember that advanced functional polymers are demanding materials. Their synthesis often begins with the synthesis of new monomers and can be complex, requiring knowledge of organic chemistry and metal catalysis. Incorporating functional monomers without deactivating their groups requires the development of new reaction conditions, catalysts, and polymerization inhibitors. The polymerization process must be carefully conducted to obtain a functional polymer while controlling the degree of polymerization. The addition of fillers must also be carefully considered. They should be introduced under conditions that will not alter their function, unless reactions between the polymer and the filler groups are planned, leading to the creation of entirely new functions.

Polymer nanocomposites (PNCs) are nanomaterials composed of nanoparticles dispersed in a polymer matrix [15,16,17]. These structures combine the properties of both components and utilize their synergistic effects to create a new material with a wide range of applications. Polymer nanocomposites are important industrial materials, widely used in packaging, energy, security systems, transportation, electromagnetic shielding, defense systems, sensors, catalysis, and information technology [18,19,20,21,22,23,24,25,26,27,28,29,30,31,32,33].

Nanomaterials are defined as materials in which at least one length dimension is less than 100 nm. Within this size range, these materials exhibit specific optical, electrical, or mechanical properties that do not occur at the macroscale.

Intensive research is being conducted to understand the physicochemical transformations of nanomaterials and nanoparticles themselves in the various environments in which products made from them will be used. Accurate physicochemical characterization of nanoparticles is essential, above all, to understand their impact on human health and the natural environment. Due to their nanoscale properties, nanostructures possess a large surface area that is electrochemically reactive and potentially capable of interacting with biological systems. Their nanosize allows them to enter the bloodstream and travel within tissues, cells, organelles, and functional biomolecular structures that would otherwise be inaccessible to larger particles. Thus, the same physicochemical properties that give them industrial utility may also confer unique toxicity in biological systems [34].

Currently, knowledge about the behavior, chemical and biological interactions, and toxicological properties of engineered nanomaterials is still limited, and certain applications remain uncertain. Industrial nanoparticle materials today constitute a tiny but significant pollution source

Nanocomposites are created by mixing nanomaterials with other materials, not necessarily at the nanometer scale, allowing the creation of materials with properties significantly different from those of the starting materials.

Expanding our understanding of functional polymers and nanocomposites and developing effective methods for their controlled production is a challenge of our times. This Special Issue is dedicated to these important topics. The aim is to explore the current state of knowledge regarding the use of functional polymers and nanocomposites in various areas of everyday life and industry, as well as their advantages and disadvantages.

This Special Issue aims to highlight the progress and fundamental aspects in the synthesis, characterization, and applications of functional polymers and nanocomposites.
